# Spatially Ordered Matrix of Nanostructured Tin–Tungsten Oxides Nanocomposites Formed by Ionic Layer Deposition for Gas Sensing

**DOI:** 10.3390/s21124169

**Published:** 2021-06-17

**Authors:** Gennady Gorokh, Natalia Bogomazova, Abdelhafed Taleb, Valery Zhylinski, Timur Galkovsky, Anna Zakhlebayeva, Andrei Lozovenko, Michael Iji, Vladimir Fedosenko, Valeri Tolstoy

**Affiliations:** 1 R&D Laboratory of Nanotechnologies, Belarusian State University of Informatics and Radioelectronics, 220013 Minsk, Belarus; gorokh@bsuir.by (G.G.); zakhlebayeva@bsuir.by (A.Z.); lozovenko@bsuir.by (A.L.); ijiolakunle2002@yahoo.com (M.I.); v.fedosenko@bsuir.by (V.F.); 2 Department of Chemistry, Technology of Electrochemical Production and Electronic Materials, Belarusian State Technological University, 220006 Minsk, Belarus; natalbogom123@gmail.com (N.B.); zhilinski@yandex.by (V.Z.); tgalkovskiy@gmail.com (T.G.); 3 Chimie ParisTech, Institut de Recherche de Chimie Paris, Paris Science & Lettres (PSL) University—CNRS, 75005 Paris, France; 4 Sorbonne Université, 75231 Paris, France; 5 R&D Laboratory of Programmable Layer-by-Layer Synthesis of Multinanolayers of Hybrid Compounds, St. Petersburg University, 199034 St. Petersburg, Russia; vptol@yandex.ru

**Keywords:** ionic layer deposition, tin-tungsten oxides, nanoporous anodic alumina matrixes, gas sensor

## Abstract

The process of layer-by-layer ionic deposition of tin-tungsten oxide films on smooth silicon substrates and nanoporous anodic alumina matrices has been studied. To achieve the film deposition, solutions containing cationic SnF_2_ or SnCl_2_ and anionic Na_2_WO_4_ or (NH_4_)_2_O·WO_3_ precursors have been used. The effect of the solution compositions on the films deposition rates, morphology, composition, and properties was investigated. Possible mechanisms of tin-tungsten oxide films deposition into the pores and on the surface of anodic alumina are discussed. The electro-physical and gas-sensitive properties of nanostructured Sn_x_W_y_O_z_ films have been investigated. The prepared nanocomposites exhibit stable semiconductor properties characterized by high resistance and low temperature coefficient of electrical resistance of about 1.6 × 10^−3^ K^−1^. The sensitivity of the Sn_x_W_y_O_z_ films to 2 and 10 ppm concentrations of ammonia at 523 K was 0.35 and 1.17, respectively. At concentrations of 1 and 2 ppm of nitrogen dioxide, the sensitivity was 0.48 and 1.4, respectively, at a temperature of 473 K. At the temperature of 573 K, the sensitivity of 1.3 was obtained for 100 ppm of ethanol. The prepared nanostructured tin-tungsten oxide films showed promising gas-sensitivity, which makes them a good candidate for the manufacturing of gas sensors with high sensitivity and low power consumption.

## 1. Introduction

In gaseous microsystems, a large group consists of chemoresistive sensors with sensitive layers based on transition metal oxides, the electrophysical surface properties of which change depending on the composition of the environment [1,2]. The mechanism of sensor chemoresistive sensitivity is based on redox reactions between gas molecules and the surface of the metal oxide sensitive layer, which leads to a change in its conductivity [3]. With the ever-growing requirements for the electrophysical characteristics of sensors, the need to analyze the minimum concentrations of individual components of complex gases and media lead to constant improvement in the design of sensors and the search for new gas-sensitive materials and their methods of deposition [4]. The most complete requirements set for selectivity, sensitivity, speed, power consumption, and cost are not satisfied by individual sensors, but by selections of thin-film sensors combined into a single microsystem [5]. The most common materials for sensitive layers are metal oxide layers based on Sn and other metals, such as Pd, Mo, W, Mn, Ni, Bi, In, and Cu [2,6], the properties and deposition methods of which are currently well studied. In this case, the mixtures of oxides or combined oxide layers, for example, SnO_2_–WO_3_, Fe_2_O_3_–ZnO, and Bi_2_WO_6_ are often used, which makes it possible to increase the sensitivity and selectivity of sensors, in turn improving their adsorption capacity and thermal stability [4,7,8]. The nanostructuring of thin films [9,10], the creation of regular arrays of nanostructures [11], as well as the formation of multicomponent metal oxide films [12] can significantly increase the functional characteristics of sensor microsystems created on their basis. At present, the well-known forming methods of nanostructured metal oxide films, using templates of nanoporous anodic alumina (NAA) [13,14,15], such as chemical deposition, electrophoretic deposition, and citrate-gel method [12,16,17], are already developed. 

The very promising chemical method for the formation of nanostructured films of various compositions is the method of ionic deposition, known as the method of successive ionic layer deposition (SILD) or the successful ionic layer adsorption and reaction (SILAR) [18]. It is based on cyclic processing of substrates in ion-containing solutions of metal salts, because of which the deposition and interaction of cations and anions occurs on the substrate surface with the formation of poorly soluble compound [19]. The most typical mechanisms for the formation of metal oxide films are the oxidation of adsorbed cations during the formation of one-component metal oxides, for example, SnO_2_, and the reduction of adsorbed anions by cations during the formation of multicomponent compounds, as in the case of Sn_x_MoO_y_ [15,20]. As shown by the first results of studies of the deposition of two-component metal oxide films on NAA matrices, such films have good regularity, reproducibility of properties, and can be used in promising photovoltaic and sensor devices [21,22].

Tin-tungsten oxides (TTO) are the most traditionally studied, because they exhibit interesting conductivity due to the presence of oxygen vacancies in the crystal lattice. Among these oxides, SnO_2−δ_ shows narrow band gap and stable electrophysical properties in a wide range of temperature from 273 K to 523 K, whereas WO_3−δ_ oxide shows interesting conductivity due mainly to the processes involving charge carriers [23,24]. At the same time, complex studies of spatially ordered tin-containing multicomponent metal oxide films by the SILAR method on structured substrates, in particular, on NAA matrices, for the nanostructured sensitive layers formation of gas sensors, have practically not been carried out. The data on the study of the specific features and mechanisms of the SILAR of metal oxides on the NAA matrices, based on the reactions of inner and outer-sphere complexes formation, are of great interest and open up the possibility of forming nanostructured films with a large active surface area, the properties of which can be controlled by varying their morphology and composition [25].

This paper presents the results of the SILAR deposition of composite films based on Sn and W oxides on the flat surfaces and in the NAA matrices, as well as the study of their morphology and elemental composition, electrophysical and gas sensitive properties of the formed structures. The complex of studies carried out made it possible to develop the scientific foundations of the technology for the manufacture of promising and stable in air atmosphere matrix-film tin-tungsten-containing oxides for sensor microsystems, providing availability, manufacturability, and precision in composition and properties.

## 2. Materials and Methods

**Figure 1:**
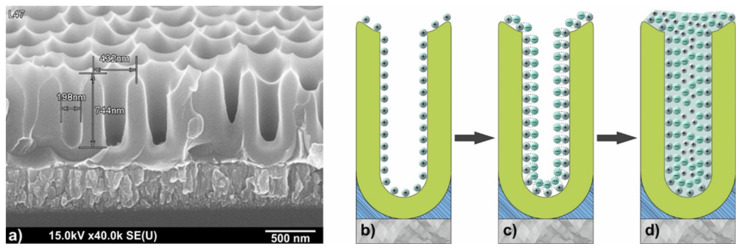
SEM image of low profile NAA matrix prepared for SILAR deposition of composite films (**a**); schematic representation of ionic deposition of tin-tungsten oxides into the pores of the NAA (**b**–**d**).

The initial samples were polished silicon substrates 100 mm in diameter, n-type, with a resistivity of 4.5 Ω cm and a crystallographic orientation (100). Silicon substrates were subjected to chemical surface treatment in a hot (348–353 K) ammonia peroxide solution, in hot (363–373 K) concentrated nitric acid, rinsing in distilled running water using squirrel brushes, followed by drying by centrifugation and dry blowing air. On the thus prepared Si substrates, magnetron deposition of aluminum with a thickness of about 1.5 µm was carried out at the substrate temperature of 503–523 K in a vacuum of 2 × 10^−4^ Pa. The sample electrochemical anodization was carried out in two stages in 0.4 M aqueous solution of tartaric acid at 295 ± 1 K, providing constant electrolyte stirring. The electrical anodizing modes were set using a Keysight N5751A system DC power supply (Keysight Technologies, Santa Rosa, CA, USA); the process parameters were recorded and monitored in situ using a Keysight 34470A digital multimeter (Keysight Technologies, Santa Rosa, CA, USA) connected via USB to a personal computer with Bench Vue software installed. Anodizing was carried out in a galvanostatic mode at current density of j = 6 mA/cm^2^. The stationary anodizing voltage was 210 V. The second anodizing was carried out under conditions identical to the first stage. The formed structure was observed in a Hitachi S-806 electron microscope (Hitachi, Japan) at an accelerating voltage of 30 kV. Figure 1a shows a SEM image of a low aspect ratio profile NAA matrix prepared for SILAR deposition of composite films.

The formation of composite metal oxide Sn_x_W_y_O_z_ films was carried out by SILAR on planar surface of monocrystalline silicon substrate, as well as on the surface of NAA matrices in cationic and anionic solutions with intermediate washes in distilled water. Before performing SILAR synthesis, the matrices were kept in distilled water at T = 363–373 K for 30 min. For the formation of deposited films of mixed tin-tungsten oxide with cationic precursor, SnF_2_ and SnCl_2_ solutions were used with the concentration of 0.003 to 0.01 M at pH 2–3. As the anionic precursor, Na_2_WO_4_, (NH_4_)_2_O·WO_3_ solutions were used with the concentration of 0.01 M at pH 6–8. The films were deposited at temperature ranging from 303 K to 333 K. The number of layering cycles was ranged from 5 to 60, after which the substrate was heated at temperature ranging from 323 K to 623 K for different time ranging from 30 to 60 min. Sequential ionic deposition of tin-tungsten oxides into the pores of the NAA matrix is schematically shown in Figure 1b–d.

The formed composite films were studied using scanning electron microscopy, Fourier transform infrared spectroscopy (FTIR) as well as a two-probe resistive method under thermodynamic and chemical exposure. Micrographs of the surface and chips of the prepared samples were obtained in a Hitachi S-806 electron microscope (Hitachi, Japan) at an accelerating voltage of 30 kV. Morphological parameters of formed structures (such as pores diameters, thicknesses of the films, grain sizes, etc.) were measured in the SEM images of surfaces and cross-sections by using the software ImageJ. Average values of morphological parameters were calculated based on measurement results. The TTO composition was investigated by IR spectroscopy, which is widely used for the analysis of thin films [26], including metal oxide composites [27]. The reflection spectra were recorded on an Thermo/Nicolet Nexus 470 FT-IR ESP Spectrometer (Thermo Fisher Scientific Inc., USA). IR Fourier spectrometer in the range 4000–400 cm^−1^ and a spectral slit width of no more than 1 cm^−1^ in the reflection and multiple disturbed total internal reflection modes. Immediately prior to spectra recording, the samples were heated in a thermostat at 393 K for desorption of condensed moisture from their surface. Purging of the spectrophotometer cell with dry air excluded the distortion of the spectra due to absorption by atmospheric moisture. Investigations of elemental compositions were performed by the electron-probe X-ray spectral microanalysis (EDX) using add-on “Bruker” for scanning electron microscope. Characteristic size of the spot from the primary ray was 2 μm. The penetration depth of the beam was from 0.1 to 2 μm. 

The study of gas sensitivity of nanostructured TTO films was performed using an experimental setup including the sealed measurement cell, the system for creating and maintaining a gas environment in the cell, and the electrical measurement devices. The measurements were performed in purified dry air with a composition of 79% nitrogen and 21% oxygen (by volume), and with a flow rate of 2 dm^3^ h^−1^. The operating temperature was the ambient air temperature (25 °C) and measured with an infrared pyrometer MASTECH MS6522B (Mastech, Shenzhen China). The experiments were monitored using the information recorded by the gas flow meters and microcontrollers, transmitted to a personal computer via the RS 485–RS 232 interface cable. The control of the measurements was performed by the specialized terminal program Hercules [28].

The sensor prepared from the tested nanostructured TTO films with a size of 3.7 × 3.7 mm was placed in the measuring cell. The measurement cycle began by turning on the system’s gas generator to create and maintain a gaseous environment. The dry air flow rate was controlled by the H250 M8 rotameter (Krohne Group, Duisburg, Germany). After a preliminary pumping, the gas was introduced into the measuring cell through the PCE 932 manometer (PCE Holding GmbH, Alicante, Spain) and the MASS-VIEW rotameter (Bronkhorst High-Tech BV, Bethlehem, PA, USA), which operated in a wide flow range from 0.01 to 500 dm^3^ min^−1^ with an accuracy of 1.5%. During the measurements, the gas concentration was gradually increased and controlled by adjusting the gas flow rate.

The resistance measurements of the prepared films were performed in a closed two-probe measuring cell in air or in active gas vapors, such as ammonia. During the measurements, the substrate temperature was varied from room temperature to 523 K using a platinum heater and was controlled by a chromel-alumel thermocouple. The resistance of the nanostructured TTO films was measured using an APPA 107 multimeter (APPA Technology Corp., Taipei, Taiwan). In addition, the measurements were repeated 5 times for each temperature and gas at different concentrations to ensure reproducibility. The operating time before the start of the measurements was 24 h at the power consumption of 60 mW in the constant heating mode. The current value was set at 35 mA from the power supply B5—49 (MIPI, Minsk, Belarus) during the heating of the sensor system. After the measurements, the cell was purged with air for 30 min to clean it. For regeneration, the samples were heated to a temperature of 723 K in dry air for 30 min [28].

## 3. Results and Discussion

### 3.1. Ionic Layer Deposition of Tin–Tungsten Oxides Nanocomposites on Silicon Substrate Planar Surface

**Figure 2:**
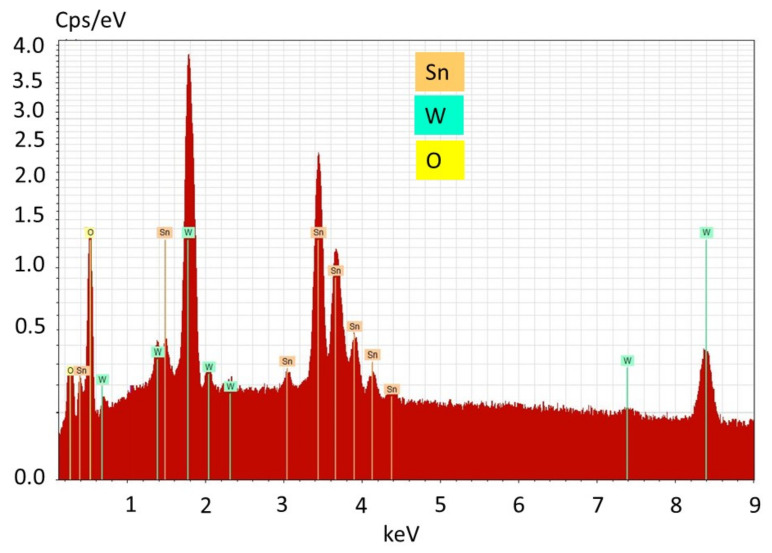
EDX spectrum of the Sn_x_WO_y_∙nH_2_O films obtained by SILAR deposition (60 cycles) with using 0.01 M SnF_2_ and 0.01 M Na_2_WO_4_.

At the first stage of the present research work, the influence of the chemical nature of anionic and cationic precursors used in the formation of thin film structures, and the growth rate and morphology of the obtained functional coatings were studied. The EDX spectrum of the thin film structures of the Sn_x_WO_y_∙nH_2_O is shown in the Figure 2. 

Table 1 shows the types of cationic and anionic solutions used in the synthesis of TTO nanocomposites, the growth rate of formed films on silicon substrates planar surfaces, as well as their thickness and elemental composition.

The tin fluoride solution was used to expand the range of operating pH values to the region of a weak acidic solution, as a result of which the reaction of oxygen with the components of the solution intensifies, and the resulting products form the thin film on the substrate surface. In a number of other experiments, tin fluoride was replaced by tin chloride, which is more resistant to hydrolysis and oxidation.

The values of the mixed oxide films thickness, obtained on the basis of electron microscopy, were in the range from 1.6 nm to 601 nm (see Table 1). The calculated values of the relative deposition rates indicate the presence of significant kinetic limitations during the deposition of films from tin chloride solution, which leads to decrease in the deposition rate using this precursor by more than two orders of magnitude as compared to the SnF_2_ solution (see Table 1). These results are in good agreement with other studies [29,30]. 

**Figure 3:**
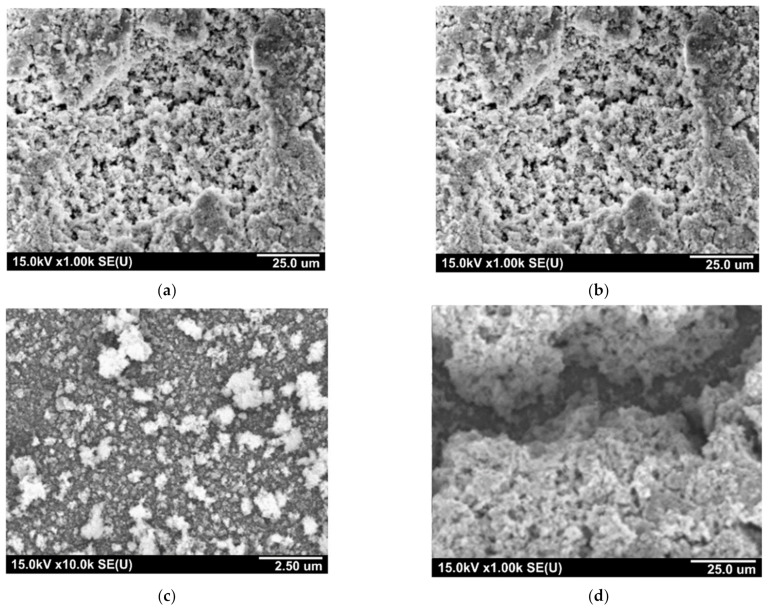
SEM images of the Sn_x_WO_y_∙nH_2_O films obtained using different precursors: (**a**) SnCl_2_; (**b**–**d**) SnF_2_; (**a**–**c**) Na_2_WO_4_; and (**d**) (NH_4_)_2_O·WO_3_.

Figure 2 shows SEM micrographs of the formed mixed oxide films on the surface of silicon substrates from different types of solutions, the compositions of which are given in Table 1. According to SEM characterization in Figure 3b,c, it can be argued that, with an increased concentration of SnF_2_ (0.01 M), the films exhibit a more developed surface morphology.

To study the effect of the anionic precursor nature on the deposited films, we replaced sodium tungstate with ammonium paratungstate, which is more stable in solution in the working pH range and easily releases the ammonium cation. However, as shown by the obtained results (see Table 1), such replacement had little effect on the deposition rate of the resulting films.

### 3.2. The Elemental Composition of Tungsten–Tin Nanocomposites

By analyzing the elemental composition of deposited films, it was shown that in contrast to the corresponding thickness, the films composition varies slightly with the precursor nature and concentration. The stoichiometric ratio of Sn:W in the prepared films was in the range from 1.5 to 2.1 (Sn_1.8 ± 0.3_WO_10.4 ± 1.2_), which corresponds to almost two-fold predominance of tin in the prepared structures (Table 1, the data of X–ray energy dispersive elemental analysis). The observed excess of oxygen content in the layers is likely a result of the significant amount of water and hydroxyl groups adsorbed on the surface of deposited films. The reduction in the concentration of SnF_2_ of about 3 times (from 0.01 to 0.0033 M) leads to the reduction of the films tin content by 20%, Sn:W ratio decreases from 1:2.0 to 1:1.6 (Table 1). The replacement of the anion precursor was shown to affect the stoichiometry of prepared mixed oxides more substantially. In particular, the use of ammonium paratungstate resulted in the reduction of the tin content and in turn the Sn:W ratio decreases from 1:2.0 to 1:1.5.

In the case of Sn_x_W_y_O_z_ film structures formation with 60 deposition cycles, different protocols of the final heat treatment were used, which included either single annealing at the temperature of 373 K (basic version) or 623 K. For deposited films after 20 cycles, the annealing temperature of 323 K was used. Furthermore, it was shown that the increase of the annealing temperature from 373 to 623 K leads to significant cracking of the deposited films, while the annealing temperature of 323 K induces the formation of less solid dense films. 

**Figure 4:**
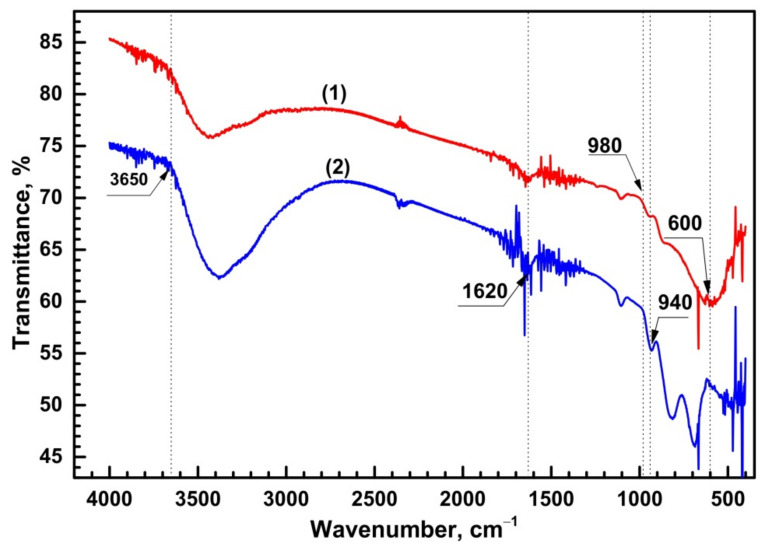
The IR absorption spectra of the Sn_x_W_y_O_z_ structure after the final heat treatment at temperatures of 623 K (1) and 373 K (2).

From the results of IR spectroscopy (Figure 4), it can be observed that an intensive removal of the water takes place, after the temperature increase of the last annealing treatment.

By increasing the annealing temperature from 373 to 623 K, one can observe that the intensity of the absorption bands located at 1640 cm^−1^ and 3650 cm^−1^, which are the characteristics of chemically and physically bound water, are significantly reduced. Absorption bands located at 600, 940 and 980 cm^−1^ can be assigned to the presence of Sn = O, W–O–Sn and W = O bonds respectively (Figure 4) [31]. The offset of the Sn = O bond oscillation frequency can be attributed to the nonstoichiometry of the prepared oxide. This result suggests that the prepared films present a heterogeneous state, characterized by the presence of both phases of individual and mixed oxides of Sn and W.

### 3.3. Ionic Layer Deposition of Tungsten–Tin Oxides Nanocomposites on Anodic Alumina Matrices

**Figure 5:**
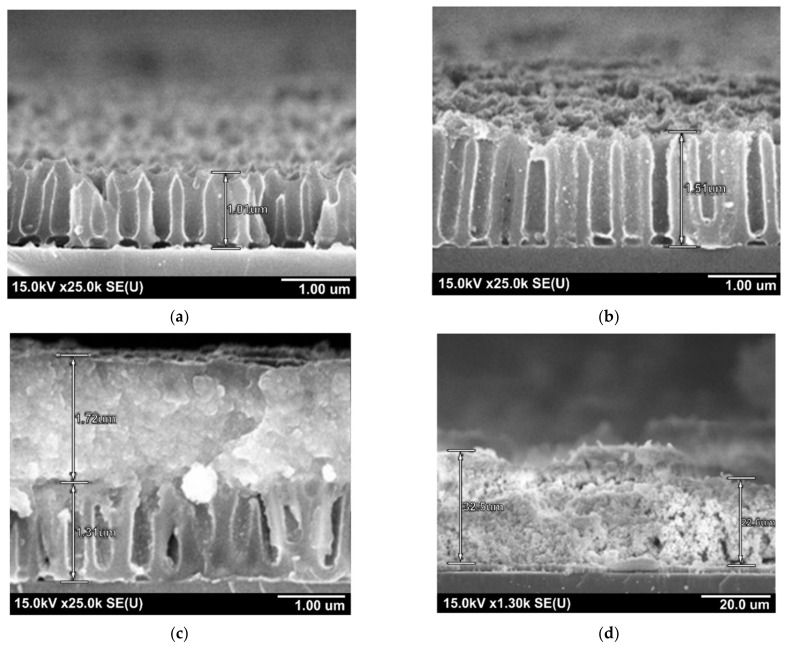
SEM images of the cross-sections of Sn_x_W_y_O_z_/Al_2_O_3_/Si systems obtained after 5 (**a**), 10 (**b**), 15 (**c**) and 30 (**d**) ion layering cycles.

The formation of NAA matrices, as well as ionic layering of Sn–W–O-containing films on them, was carried out according to the methods described in Section 2. In this series of experiments, precursor’s solutions of SnF_2_ (0.01 M) and Na_2_WO_4_ (0.01 M) were used, and the number of deposition cycles varied from 5 to 30. SEM photographs of the TTO nanocomposites cross-sections formed after 5, 10, 15, and 30 layer-by-layer synthesis cycles are shown in Figure 5. As can be seen from the above images, after 5 cycles, a slightly noticeable rash appeared on the surface of the NAA matrices, while the total film thickness did not increase. After 10 cycles, distinguishable particles of the deposited material have already formed inside the pores and on the surface of the matrix, although the increase in the thickness of the total film is quite insignificant. After the next 15 cycles of ionic layering, the film covers the pore walls and completely fills the pores of NAA matrices, forming a continuous film on its surface. The SEM micrographs shown in the Figure 5 shows that the thickness of the films formed at different number of cycles is not proportional to their number. This is especially noticeable when using NAA matrices. For example, the thickness of TTO films after 60 deposition cycles on flat silicon substrates is comparable to the thickness of films deposited after 30 cycles on NAA matrices, while the relative deposition rate on flat substrates is almost 1.8 times lower than on nanoporous matrices.

**Figure 6:**
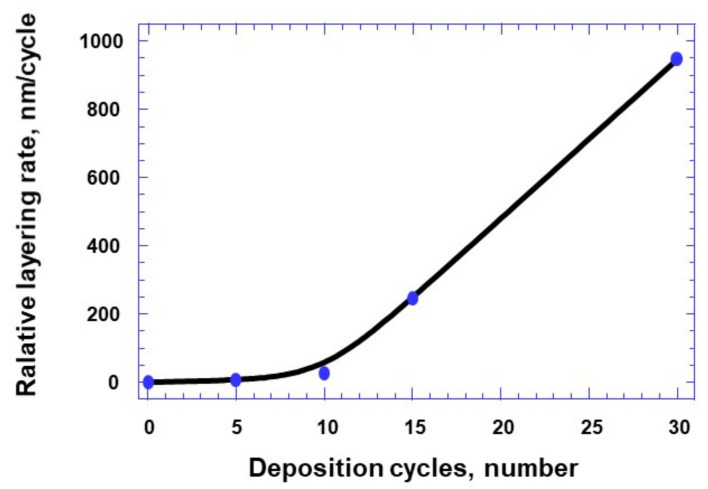
The dependence of the average speed of the Sn_x_W_y_O_z_ film layering on the number of processing cycles.

In addition, it was found that the sedimentation rate in the first 10 cycles is much lower than in the subsequent ones. The formation of local bottom and surface deposition centers occurs with an increase in the number of deposition cycles from 5 to 10 (Figure 6). In addition, it was found that the formation of a continuous layer on the surface of the NAA matrix occurs with a predominance of the surface deposition mechanism along the horizontal plane with an increase in the number of deposition cycles from 10 to 15. Surface deposition on top of the matrix and insignificant near-wall deposition on vertical walls and bottom deposition is probably associated with a change in the mechanism of layer formation from the ionic to ionic-colloidal deposition mechanism [23]. This assumption makes it possible to explain the significant thickness of the deposited layer on the matrix surface (more than 1 μm) when the number of deposition cycles is more than 15. The dependence of deposited HTO layer thickness on the number of deposition cycles has two linear sections, the first one is gentle up to the 10th cycle, and the second one is steep from 10 to 30 cycles (Figure 6). These data differ from the results presented in the literature by different authors for the SILAR method [16,23], whereby the dependence of the growth of the deposited layer thickness on the number of cycles is linear.

For all deposited films, the ratio of Sn:W was close to 2.0, and the tin content increases with the number of deposition cycles. These results can be useful in the framework of the preventive approach implementation against the colloidal deposition mechanism of tin-containing particles in the case of cationic precursor and the ion deposition mechanism of polyvalent ions in the case of anionic precursor.

### 3.4. Electrophysical Characteristics of TTO Nanocomposites

**Figure 7:**
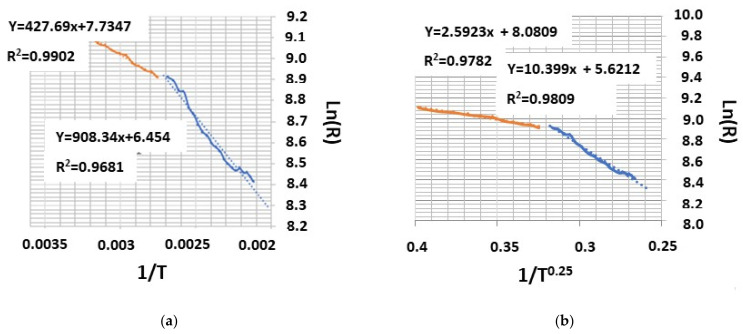
Temperature dependence of electrical resistance for nanostructured TTO on NAA as: (**a**) lnR = f(1/T); (**b**) lnR = f(1/T^0.25^).

To assess the electrical properties of spatially ordered TTO matrices, temperature studies of their electrical conductivity were carried out. Preliminary studies of the electronic transport properties have shown that the formed TTO nanocomposites have a negative temperature dependence of resistivity, as in conductive materials. In this case, the resistance of the films formed after 15 cycles of Sn_x_W_y_O_z_ deposition on smooth surface of Si substrates at room temperature was about 11 MΩ, while the resistance of nanostructured Sn_x_W_y_O_z_ films after 15 cycles of deposition on the surface of NAA matrices was 20 MΩ, which is approximately 1.8 times higher than on smooth Si surface. This is due to the structured film surface and unequal thickness throughout its entire length along the substrate. When heated to 523 K, the resistance in the TTO/Si system decreased to 4.2 MΩ, approximately by 2.6 times, while the resistance of the nanostructured TTO/NAA decreased to 4.6 MΩ, i.e., 4.35 times. With cooling, the resistance increased, but did not reach the initial value, and amounted to about 90% of the initial value. After heating and cooling, the nanostructured film showed a high stability of the resistance change. In this case, the calculated temperature coefficient of resistance in this temperature range was 1.6 × 10^−3^ K^−1^. On the temperature dependence of the nanostructured Sn_x_W_y_O_z_ layer on NAA, two regions can be distinguished, namely a low-temperature part from 293 to 343 K and a high-temperature part from 343 to 523 K. After the approximation, this dependence was plotted in the following coordinates lnR = f (1/T) and lnR = f (1/T^0.25^) (Figure 7). As can be seen from these dependences, a more reliable approximation of the low-temperature section is observed in the coordinates lnR = f (1/T), while the high-temperature section is more reliably approximated in the coordinates lnR = f (1/T^0.25^). This result can probably be associated with change in the mechanism of the electrical transfer in this system from the band mechanism in the low-temperature region to the hopping mechanism in the high-temperature region, in which electroactive defects of the nanostructure participate in the electrical conductivity.

In the temperature range from 293 to 343 K, the calculated activation energy of conductivity was 11.9 meV, and in the temperature range from 343 K to 523 K–69.6 meV, i.e., for the high-temperature region, the activation energy is five times higher than for the low-temperature region, which can be associated with the activation of hopping conductivity in the band gap of the mixed nonstoichiometric oxide Sn_x_W_y_O_z_.

### 3.5. Gas Sensitive Properties of TTO Nanocomposites

**Figure 8:**
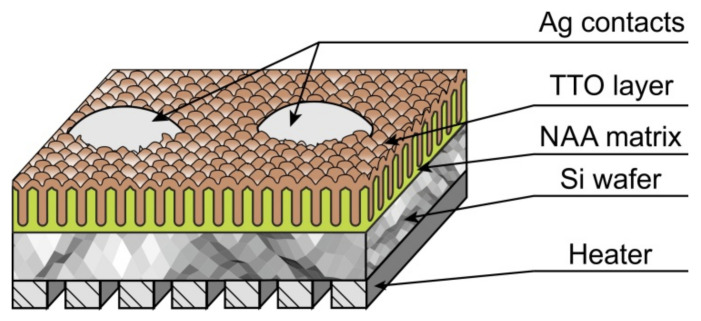
The schematic representation of the test sensory structure.

To study the gas-sensitive properties of nanostructured TTO films, test structures were fabricated, which are silicon substrate with NAA on which compound of Sn_x_W_y_O_z_ were deposited by the 15 cycles SILAR method. To give the film a crystalline structure, it was annealed in vacuum at temperature of 823 K for 40 min. On the backside, there was a platinum heater providing a stable temperature regime during measurements. Contacts were formed on the surface of the film under study to register the change in resistance as result of chemisorption of the analyzed gas molecules with the film surface. The schematic representation of the test sensory structure is shown in Figure 8.

**Figure 9:**
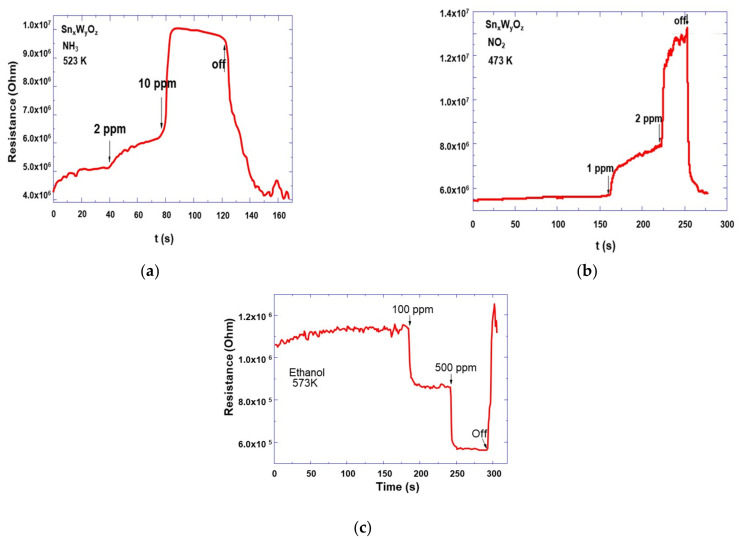
The responses of the TTO films on NAA matrixes: (**a**) to 2 and 10 ppm of ammonia at 523 K; (**b**) to 1 and 2 ppm of nitrogen dioxide at 473 K; (**c**) to 100 and 500 ppm of ethanol at 523 K.

The responses of the TTO films to ammonia, nitrogen dioxide, carbon monoxide, and ethanol were analysed at operating temperatures between 423 K, and 523 K. The heater provided heating of the test sensor structure in this temperature range at a power consumption of 29–42 mW. During the measurements, the sensor structure was maintained at working temperature in the measuring chamber when purified air was passed through it. Figure 8 presents the experimentally measured responses of the prepared test structures to low concentrations of ammonia (Figure 9a), nitrogen dioxide (Figure 9b), and ethanol vapors (Figure 9c). Sensory structures with TTO films showed the highest sensitivity to ammonium at temperature of 523 K. Within 20 s after adding of 2 ppm ammonia to the air, the structure reacted, the resistance increased to 6.2 MΩ. Then, after the introduction of 10 ppm ammonia, the resistance has grown to 10 MΩ. The sensitivity for analysed gases calculated using the relation S = (R_gas_ − R_air_)/R_air_. Thus, the test structures showed the high sensitivity to ammonia, the responses to concentrations of 2 and 10 ppm were 0.35 and 1.17, respectively (Figure 9a).

The tin-tungsten oxide nanocomposites have also been shown to be highly sensitive to nitrogen dioxide. At the same time, the operating temperatures for this gas lay in a wider range from 420 to 525 K, and the sensitivity increased slightly with increasing temperature. Therefore, we chose a lower heating level of the test structure in order to ensure the minimum power consumption at an acceptable sensitivity at NO_2_ extremely low concentrations. Figure 9b shows responses to nitrogen dioxide at 473 K. The test structure sensitivity with TTO nanostructured active layer at nitrogen dioxide concentrations of 1 and 2 ppm were 0.48 and 1.4, respectively, at temperature of 473 K (Figure 9b).

As for other analyzed gases, the sensor structures showed a barely noticeable response to carbon monoxide in the given temperature range, and it was not possible to calculate its sensitivity. Nevertheless, for ethanol vapors, the sensors also showed high sensitivity, but at higher operating temperatures. At 100 ppm ethanol the sensitivity was 0.868 at 573 K (Figure 9c). The studied sensor structure was distinguished by high stability of measurements, in particular, after five measurements of responses in the analyzed gas medium, the initial and final resistances differed by less than 10%.

The use of modified porous anodic alumina films as support material for forming continuous tin-tungsten oxide layers with high surface area will be of vast practical importance for the fabrication of new gas sensors having the higher sensing area and the higher surface-to-volume ratio. It is readily expected that the new sensors will have superior selectivity and sensitivity as compared to the traditional gas sensors in the literature [32,33,34,35]. Additionally, and importantly, the approach and technique developed here should be of interest for preparing anodic alumina matrixes and templates, on various types of substrates, for the direct deposition of pure metals and semiconductors for many other functional utilizations in the fields of modern micro- and nanoelectronics [32,33,34,35].

## 4. Conclusions

Using the SILAR deposition method and different solutions of precursors, it was shown that a preparation of different mixed tin tungsten oxides by the SILAR deposition of Sn_x_W_y_O_z_ on flat Si and nonporous anodic alumina templates was successfully achieved. It was also demonstrated that the deposited film thickness ranged from 50 nm to 30 µm when the number of deposition cycles was increased from 10 to 60. A colloidal deposition growth model of tin—containing particles in the cationic precursor of SnF_2_ and co-deposition of tungsten-containing particles in the anionic precursor of Na_2_WO_4_ or (NH_4_)_2_O·WO_3_ was proposed to explain the formation of micro-nanoscale structure of prepared films. A predominance of tin with approximate composition of Sn_1.8±0.3_WO_10.4±1.2_ was observed with the use of cationic precursors. Furthermore, the prepared mixed oxide was shown to have different propensity to hydrolysis, e.g., SnF_2_ or SnCl_2_. The prepared TTO structures exhibit stable semiconductor properties characterized by high resistance and low temperature coefficient of electrical resistance of about 1.6 × 10^−3^ K^−1^. The TTO films showed high sensitivity to ammonia, with responses to concentrations of 2 and 10 ppm at 523 K were 0.35 and 1.17, respectively. However, the sensitivity of prepared TTO nanostructured active layer to nitrogen dioxide concentrations of 1 and 2 ppm were 0.48 and 1.4, respectively, at a temperature of 473 K. As for other gases, the sensor structures showed a barely noticeable response to carbon monoxide in the given temperature range, and it was not possible to calculate its sensitivity. Nevertheless, for ethanol vapors, the sensors also showed high sensitivity, but at higher operating temperatures of 573 K. The studied sensor structure was distinguished by a high stability of measurements, in particular, after five measurements of responses in the analyzed gas medium, the initial and final resistances differed by less than 10%. 

The use of modified porous anodic alumina films as support material for forming continuous tin-tungsten oxides layers will be of vast practical importance for the fabrication of new gas sensors having a higher sensing area and in turn higher sensitivity.

## Figures and Tables

**Table 1:** Characteristics of Sn_x_W_y_O_z_ thin film structures formed from various precursors on silicon substrates planar surfaces.

Precursor Parameters		Thickness of the TTO Layer, nm	Relative Layering Rate, nm per Cycle	Elemental Composition of the TTO Films According to the Data of X-ray Energy Dispersive Elemental Analysis	
				Element	Atomic Content, %
SnCl_2_ Na_2_WO_4_	0.003 M pH = 3.5 0.01 M pH = 8	48 (30 cycles)	1.6	O	80.41
				Sn	12.17
				W	4.07
				Cl	1.10
				Na	2.25
SnF_2_ Na_2_WO_4_	0.003 M pH = 3.5 0.01 M pH = 8	556 (60 cycles)	9	O	72.12
				Sn	15.14
				W	11.51
				F	1.71
				Na	0.84
SnF_2_ Na_2_WO_4_	0.01 M pH = 3 0.01 M pH = 8	3200 (60 cycles)	533	O	76.19
				Sn	12.95
				W	7.49
				F	0.91
				Na	0.34
SnF_2_ (NH_4_)_2_O·WO_3_	0.01 M pH = 3 0.01 M pH = 3	3600 (60 cycles)	601	O	75.45
				Sn	14.12
				W	9.67
				Cl	0.56
				Na	0.43

## Data Availability

Not applicable.
